# Natural Infection of *Nyssorhynchus darlingi* and *Nyssorhynchus benarrochi* B with *Plasmodium* during the Dry Season in the Understudied Low-Transmission Setting of Datem del Marañon Province, Amazonian Peru

**DOI:** 10.4269/ajtmh.23-0058

**Published:** 2023-06-26

**Authors:** Jan E. Conn, Sara A. Bickersmith, Marlon P. Saavedra, Juliana A. Morales, Freddy Alava, Gloria A. Diaz Rodriguez, Clara R. del Aguila Morante, Carlos G. Tong, Carlos Alvarez-Antonio, Jesus M. Daza Huanahui, Joseph M. Vinetz, Dionicia Gamboa

**Affiliations:** ^1^ Wadsworth Center, New York State Department of Health, Albany, New York;; ^2^ Department of Biomedical Sciences, School of Public Health, State University of New York-Albany, Albany, New York;; ^3^ Amazonian International Center of Excellence for Malaria Research, Laboratorios de Investigación y Desarrollo, Facultad de Ciencias y Filosofía, Universidad Peruana Cayetano Heredia, Lima, Peru;; ^4^ Ministry of Health, Iquitos, Peru;; ^5^ Laboratorio de Salud Pública-Gerencia Regional de Salud de Loreto, GERESA, Iquitos, Peru;; ^6^ Gerencia Regional de Salud de Loreto, GERESA, Iquitos, Peru;; ^7^ Red de Salud Datem del Marañon – Gerencia Regional de Salud de Loreto, GERESA, Iquitos, Peru;; ^8^ Laboratorio de Malaria: Parásitos y Vectores, Laboratorios de Investigación y Desarrollo, Facultad de Ciencias y Filosofía, Universidad Peruana Cayetano Heredia, Lima, Peru;; ^9^ Section of Infectious Diseases, Department of Internal Medicine, Yale School of Medicine, New Haven, Connecticut;; ^10^ VA Connecticut Healthcare System, West Haven, Connecticut;; ^11^ Instituto de Medicina Tropical Alexander von Humboldt, Universidad Peruana Cayetano Heredia, Lima, Peru

## Abstract

The persistence of malaria hotspots in Datem del Marañon Province, Peru, prompted vector control units at the Ministry of Health, Loreto Department, to collaborate with the Amazonian International Center of Excellence for Malaria Research to identify the main vectors in several riverine villages that had annual parasite indices > 15 in 2018–2019. Anophelinae were collected indoors and outdoors for two 12-hour nights/community during the dry season in 2019 using human landing catch. We identified four species: *Nyssorhynchus benarrochi* B, *Nyssorhynchus darlingi*, *Nyssorhynchus triannulatus*, and *Anopheles mattogrossensis*. The most abundant, *Ny*. *benarrochi* B, accounted for 96.3% of the total (7,550/7,844), of which 61.5% were captured outdoors (4,641/7,550). Six mosquitoes, one *Ny. benarrochi* B and five *Ny. darlingi*, were infected by *Plasmodium falciparum* or *Plasmodium vivax*. Human biting rates ranged from 0.5 to 592.8 bites per person per hour for *Ny. benarrochi* B and from 0.5 to 32.0 for *Ny. darlingi*, with entomological inoculation rates as high as 0.50 infective bites per night for *Ny. darlingi* and 0.25 for *Ny. benarrochi* B. These data demonstrate the risk of malaria transmission by both species even during the dry season in villages in multiple watersheds in Datem del Marañon province.

## INTRODUCTION

In Peru, many malaria cases (∼84% of 26,621 in 2022)^1^ originate in the hypoendemic region of Loreto Department.^2^^,^^3^ One indicator of the health burden due to malaria is the measure of the economic burden of productivity loss, calculated using the disability-adjusted life year and the gross domestic product. A recent study based on Peruvian data determined that whereas the economic burden of productivity loss for Loreto from 2001 to 2019 was several times that of Peru overall, in 2019 alone it was estimated to be 30 times higher, a stark reminder of the disproportionate effect of health disparities in this region.^4^ Locally and globally, malaria is heterogeneous owing to differences in landscape, vector distribution and ecology, and human behavior, among other factors,^5^^,^^6^ and in Loreto many riverine villages are transmission hotspots.^7^ Malaria endemic regions in several parts of Amazonian Peru have been identified and investigated, yet the province of Datem del Marañon remains understudied.

Aside from well-known annual seasonal mosquito abundance and malaria cycles (high during the rainy season and low during the dry season), malaria case numbers in Amazonian Peru have a history of fluctuation.^8^ Despite a marked reduction in cases associated with the major control initiative “Control de la Malaria en las Zonas Fronterizas de la Región Andina: Un Enfoque Comunitario – PAMAFRO (2006–2011), by 2012 case levels began to increase,^9^ reaching epidemic proportions in many communities by 2017.^10^ Notably, case numbers since 2017 declined by an estimated 50–60%,^11^^,^^12^ attributed mainly to the Malaria Zero Program (MZP), put into place in 2017 by the Peruvian government.^4^^,^^13^ The MZP control activities have included free antimalarials, test-and-treat strategies, larviciding, and pyrethroid spray. Nevertheless, during epidemiological weeks 1–31 in Peru in 2022, the number of cases reported was 15,811, a major increase from 2021 data, when 9,698 cases were reported during the same period.^1^

Within Loreto, the principal malaria vector is *Nyssorhynchus darlingi*, a remarkably adaptable species that invaded Iquitos, Peru in the 1990 s^14^^–^^16^ and continues to dominate malaria transmission throughout much of the Amazon region of northern South America.^17^^,^^18^ However, prior to the reinvasion of *Ny. darlingi* into Loreto in the 1990s, *Nyssorhynchus benarrochi* was considered to be the main vector, especially in some areas of western Loreto and eastern Peru.^19^^–^^21^ Based on distribution and molecular taxonomy, it is probable that the species previously known as *Ny*. *benarrochi* in Peru is actually *Nyssorhynchus benarrochi* B,^22^^,^^23^ a member of a species complex that comprises *Nyssorhynchus benarrochi* s.s., *Ny. benarrochi* B, *Nyssorhynchus benarrochi* G1, and *Nyssorhynchus benarrochi* G2.^24^ The current known distribution of *Ny. benarrochi* B, aside from Peru, includes southern Colombia,^25^ Amazonian Ecuador,^23^ and both western (Acre state) and eastern (Para state) Amazonian Brazil.^24^^,^^26^ We hypothesized a role for *Ny. benarrochi* B in malaria transmission in Datem del Marañon based in part on reported high anthropophily and infection with *Plasmodium* in eastern Peru and southern Colombia,^21^^,^^27^ although there have also been instances where *Ny. benarrochi* is abundant and highly anthropophilic but not detected as infected.^28^

Knowledge of biting behavior is an essential part of understanding transmission risk, and such information provides insights that can be used to improve surveillance and intervention.^29^^,^^30^ Rarely, if ever, have peak biting time and location been investigated in *Ny. benarrochi* B, and it is not known whether or to what extent its biting activity is influenced by environmental variables including indoor residual spray, long-lasting insecticidal bed net use, or human sociodemographics. Intensity of malaria transmission can be estimated by use of the entomological inoculation rate (EIR). In the Amazon Basin, the main technique to calculate *Plasmodium* infectivity of mosquitoes remains ELISA,^31^ although molecular methods such as nested polymerase chain reaction (PCR)^32^ and real-time PCR of the small subunit of the 18S ribosomal RNA (rRNA) have become more common.^33^^,^^34^ The EIR is useful for evaluation and comparison of the effectiveness of vector interventions across landscapes and during malaria elimination efforts. It is also considered to be one of the best metrics for measuring malaria transmission that can be incorporated into the development of model-predicted maps to accurately pinpoint locations of malaria transmission risk, particularly in low-transmission settings during malaria elimination efforts, and where asymptomatic infections are not detected by more traditional surveillance systems.^35^

Datem del Marañon is one of eight provinces that together comprise Loreto Department. Although large (46,000 km^2^), it is sparsely populated, with an estimated one inhabitant/km^2^. From 2000 to 2017, high transmission of *Plasmodium falciparum* and *Plasmodium vivax* characterized Datem del Marañon, along with other northwestern provinces in Loreto.^36^ Perhaps because of its remoteness and low population, Datem del Marañon has not been the focus of any systematic vector biology studies. The objective of this study was to identify the malaria vectors in this region of Datem del Marañon in Amazonian Peru and to calculate the entomological indices to estimate the risk of local malaria transmission.

## MATERIALS AND METHODS

### Study region.

**Figure 1.**
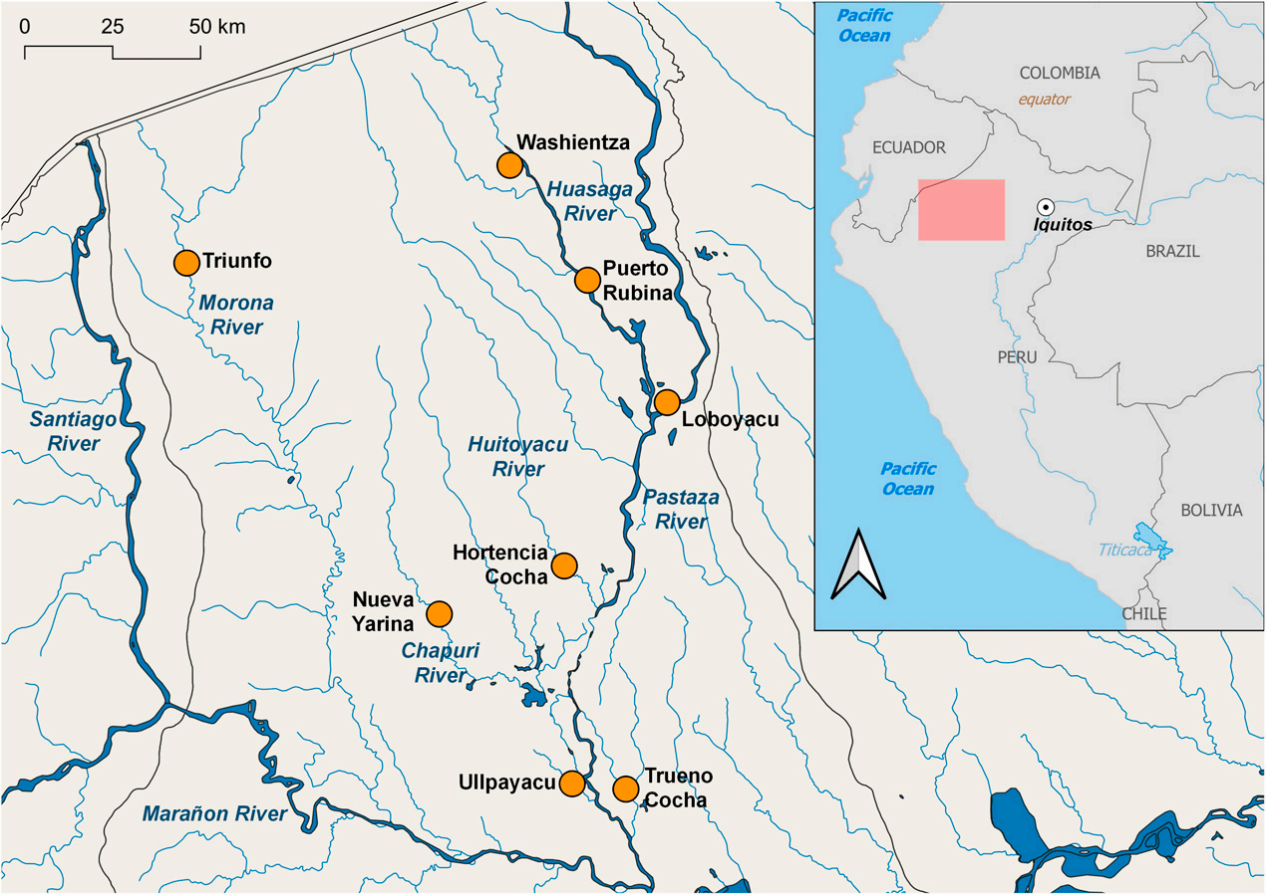
Map of mosquito collection localities within the Datem del Marañon province, Loreto Department, Amazonian Peru.

Loreto Department is characterized by a distinctive rainy season (November–May) and a dry season (June–October), although rainfall (cumulative average of 2,500 mm) occurs throughout the year. The human population of 883,510 is distributed among cities and large and small villages.^35^ Datem del Marañon province is mainly a tropical rainforest climate according to the Köppen climate classification.^37^ The major rivers are the Marañon, Pastaza, Huasaga, and Morona (Figure 1). The capital, San Lorenzo de Loreto, situated on the northern banks of the Marañon River has a population of 8,216 (Peruvian census 2017) and is accessible by river or small aircraft.^38^ Most inhabitants are engaged in swidden-fallow agroforestry, cultivation of crops in the floodplains and exposed riverbeds, extraction of forest products, fishing, and hunting.^39^ Crops include rice, cowpea, plantain, manioc, and maize.^40^ In the six main study villages, the annual parasite indice (API; the number of confirmed new malaria cases registered in a specific year per 1,000 individuals under surveillance) ranged from 18 in the largest village of Ullpayacu (population 1,716) to 2,398 in the village of Hortencia Cocha with 83 inhabitants (Table 1).

**Table 1:** Details of collection localities within the province of Datem del Marañon where mosquito collections occurred, human population numbers, annual human malaria case estimates, and API

Locality	District	Latitude	Longitude	Population	2018 Cases		API	2019 Cases		API	2020 Cases		API
					Pf	Pv		Pf	Pv		Pf	Pv	
Washientza	Andoas	−3.064560	−76.757360	604	227	502	1,207	180	394	950	132	280	682
Loboyacu	Andoas	−3.670834	−76.355690	301	23	80	332	6	13	63	7	9	53
Hortencia Cocha	Andoas	−4.088269	−76.618550	83	75	124	2,398	80	98	2,145	87	43	1,566
Nueva Yarina	Pastaza	−4.211420	−76.937980	122	24	230	2,082	22	127	1,221	42	70	918
Ullpayacu	Pastaza	−4.644330	−76.598210	1,716	5	93	57	2	29	18	10	25	20
Trueno Cocha	Pastaza	−4.658039	−76.461150	253	2	70	285	0	39	154	9	59	269
Totals	–	–	–	–	356	1,099	–	290	700	–	287	486	–

API = annual parasite index; Pf = *Plasmodium falciparum*; Pv = *Plasmodium vivax.*

### Mosquito sampling.

Mosquitoes were collected from three localities within the Pastaza district (Ullpayacu, Trueno Cocha, and Nueva Yarina) and three within the Andoas district (Hortencia Cocha, Loboyacu, and Washientza) of Datem del Marañon province (Figure 1, Table 1). Collections were conducted in 2019 indoors and outdoors (peridomestic, within approximately 10 m of the main house entrance) using human landing catch (HLC) by two persons/locality for 12 hours from 18:00 to 06:00, for two nights/locality as follows: Nueva Yarina and Hortencia Cocha, August 3–5; Washientza and Ullpayacu, August 5–7; and Loboyacu and Truena Cocha, August 7–9.

Mosquito collecting including HLC is conducted as part of the routine work of field personnel at Laboratorio de Salud Pública-Gerencia Regional de Salud de Loreto, GERESA, Peru, and, as such, is considered a safety management issue. All field personnel are trained in the safe and responsible collection of mosquitoes and other vectors that might transmit pathogens.

We also examined two samples of anophelines collected during 4-hour collections (18:00–22:00) by HLC indoors and outdoors from Puerto Rubina on April 10, 2021 and Triunfo on April 25, 2021 (Figure 1), confirmed the Anophelinae species identification, and tested these specimens for *Plasmodium*. These secondary collections were not used to calculate any entomological indices.

### Testing for *Plasmodium.*

A total of 7,827 mosquitoes, using heads and thoraces, in pools of one to eight mosquitoes based on capture time, species (*Ny. benarrochi* B or *Ny. darlingi*), and specific locality were tested at the Universidad Peruana Cayetano Heredia in Lima, Peru, for detection of *Plasmodium* infection with an ELISA as in Saavedra et al.^41^ To calculate the infection rate (IR) for each Anophelinae species (IR = # mosquitoes infected with *Plasmodium/*# mosquitoes of the same species tested) and the EIR = HBR × IR, each of the positive pools was conservatively estimated to include one positive mosquito. The human biting rate (HBR) was calculated as the mean number of mosquitoes collected by HLC per person per night.

At the Vector Biology and Population Genetics Laboratory at the Wadsworth Center, New York State Department of Health in Albany, New York, genomic DNA was extracted from each individual abdomen (Qiagen DNeasy Blood & Tissue Kit, Germantown, MD) of the 33 specimens that comprised the positive (*N* = 6) and possible positive (*N* = 3) pools for ELISA and was tested for *Plasmodium* species infection using 18S rRNA, in duplicate, with a triplex real-time PCR.^33^ The possible positives were below the optical density (OD) but close to the average of twice the OD of the negative controls, although the real-time PCR tests of abdomens were negative. We used only the six positive ELISA results to calculate the entomological indices. Most vectors in malaria endemic Peru have relatively low infectivity rates; consequently, abdomens may contain very low titers of *Plasmodium*. Despite the higher sensitivity of real-time PCR, it is not uncommon to find a discrepancy between the two results, that is, more positive or possible positive pools from ELISA versus real-time PCR.

### Morphological and molecular species identification.

All captured mosquitoes were identified initially using regional morphological keys^42^^,^^43^ at the Laboratorio de Salud Publica-Gerencia Regional de Salud de Loreto, DIRESA in Iquitos, Peru. The 2021 mosquito samples from Puerto Rubina and Triunfo, as well as individual *Ny. benarrochi* B and *Ny. darlingi* from the positive ELISA pools, were identified molecularly for species confirmation using a PCR of the ribosomal internal transcribed spacer 2 (ITS2) region, followed by a double-digest restriction fragment length polymorphism (RFLP).^44^ The cytochrome c oxidase subunit I (COI) barcode region was amplified^45^ for all mosquito samples that could not be identified by the ITS2-PCR-RFLP patterns and sent for Sanger sequencing in the forward direction only at the Advanced Genomic Technologies Core (Wadsworth Center). Raw sequences were cleaned, edited, and checked for stop codons and pseudogenes using the platform Geneious Prime.^46^ Sequences were queried for species match in the Barcode of Life Data System (www.barcodinglife.org) or GenBank (https://www.ncbi.nlm.nih.gov/genbank/). Following GenBank protocol, 16 sequences of *Anopheles* *benarrochi* B, one *Anopheles darlingi*, three *Anopheles tadei*, and one *Anopheles mattogrossensis* (21 total) from this study have been deposited under accession numbers OP964656–OP964676.

## RESULTS

### Vector biology.

**Figure 2.**
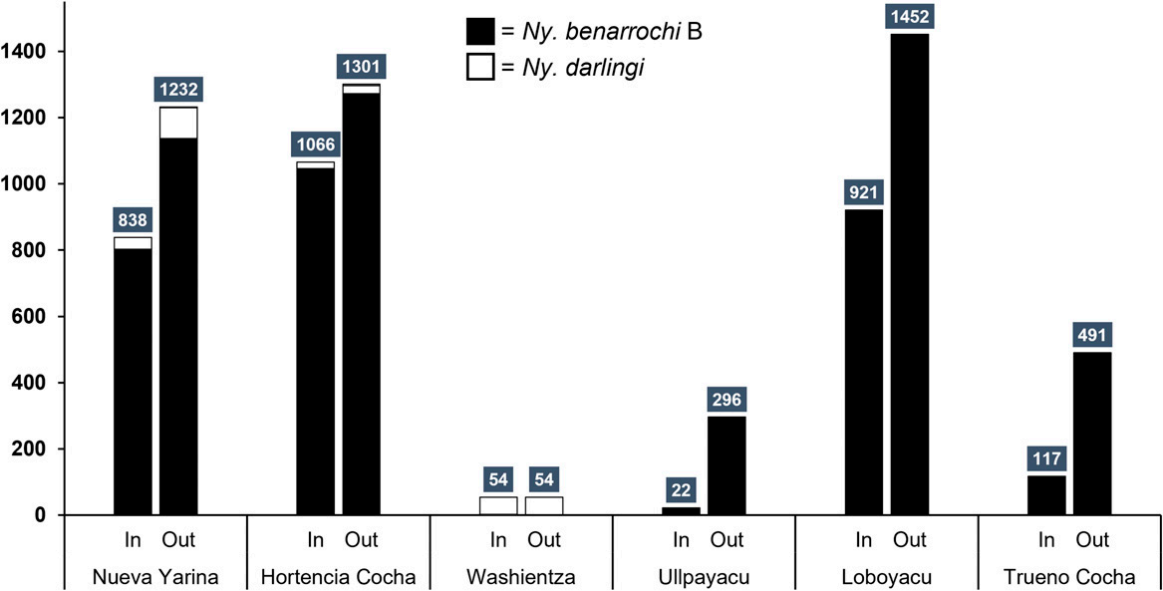
Total number (counts) of *Nyssorhynchus benarrochi* B and *Nyssorhynchus darlingi* captured indoors (in) or outdoors (out) in Datem del Marañon, Peru, in 2019 by locality.

A total of 7,844 Anophelinae mosquitoes were captured across the six main collection localities. Species detected were *Ny. benarrochi* B, *Ny. darlingi, Nyssorhynchus triannulatus* s.l., and *An. mattogrossensis.* The most abundant species captured was *Ny. benarrochi* B (*N* = 7,550) in all but one locality (Washientza), where *Ny. darlingi* was the most abundant, albeit at low numbers (Table 2, Figure 2). The samples captured in 2021 in Puerto Rubina were all *Ny. darlingi* (*N* = 18); in contrast, of the 19 mosquitoes collected in Triunfo, nine were *An. mattogrossensis*, four were *Ny. darlingi*, three were *Nyssorhynchus tadei*, a recently named species in the Oswaldoi-Konderi complex,^47^ and two were *Ny. benarrochi* B. One could not be identified (degraded DNA).

**Table 2:** Number of mosquitoes collected by species, indoors and outdoors, per locality in the province of Datem del Marañon, HBR, IR, and EIR

Locality	Species	Indoor *N*	Outdoor *N*	Total *N*	HBR (± SE)	# Infected		IR	EIR
						Pv	Pf		
Washientza	*Ny. benarrochi* B	2	0	2	0.5 (0.5)	–	–	0.0000	0.000
	*Ny. darlingi*	52	54	106	26.5 (8.5)	1	–	0.0094	0.250
	*An. mattogrossensis*	0	0	0	0 (0)	–	–	N/A	N/A
	*Ny. triannulatus* s.l.	0	0	0	0 (0)	–	–	N/A	N/A
	Total	54	54	108	–	–	–	–	–
Loboyacu	*Ny. benarrochi* B	921	1,450	2,371	592.8 (308.3)	1	–	0.0004	0.250
	*Ny. darlingi*	0	0	0	0 (0)	–	–	N/A	N/A
	*An. mattogrossensis*	0	2	2	0.5 (0.5)	–	–	0.0000	0.000
	*Ny. triannulatus* s.l.	0	0	0	0 (0)	–	–	N/A	N/A
	Total	921	1,452	2,373	–	–	–	–	–
Hortencia Cocha	*Ny. benarrochi* B	1,046	1,272	2,318	579.5 (19)	–	–	0.0000	0.000
	*Ny. darlingi*	20	25	45	11.3 (5.3)	1	1	0.0444	0.500
	*An. mattogrossensis*	0	4	4	1 (0)	–	–	0.0000	0.000
	*Ny. triannulatus* s.l.	0	0	0	0 (0)	–	–	N/A	N/A
	Total	1,066	1,301	2,367	–	–	–	–	–
Nueva Yarina	*Ny. benarrochi* B	803	1,137	1,940	485 (199)	–	–	0.0000	0.000
	*Ny. darlingi*	35	93	128	32 (6.5)	–	2	0.0156	0.500
	*An. mattogrossensis*	0	2	2	0.5 (0)	–	–	0.0000	0.000
	*Ny. triannulatus* s.l.	0	0	0	0 (0)	–	–	N/A	N/A
	Total	838	1,232	2,070	–	–	–	–	–
Ullpayacu	*Ny. benarrochi* B	22	295	317	79.3 (67.3)	–	–	0.0000	0.000
	*Ny. darlingi*	0	0	0	0 (0)	–	–	N/A	N/A
	*An. mattogrossensis*	0	1	1	0.3 (0.3)	–	–	0.0000	0.000
	*Ny. triannulatus* s.l.	0	0	0	0 (0)	–	–	N/A	N/A
	Total	22	296	318	–	–	–	–	–
Trueno Cocha	*Ny. benarrochi* B	115	487	602	150.5 (34.5)	–	–	0.0000	0.000
	*Ny. darlingi*	1	1	2	0.5 (0.5)	–	–	0.0000	0.000
	*An. mattogrossensis*	0	3	3	0.8 (0.3)	–	–	0.0000	0.000
	*Ny. triannulatus* s.l.	1	0	1	0.3 (0.3)	–	–	0.0000	0.000
	Total	117	491	608	–	–	–	–	–

*An*. = *Anopheles*; EIR = the number of infective bites per person per 12-hour night; HBR = the average bites per person per night (b/p/n) calculated from a mean of two nights/12 hours per night per locality; Indoor *N* = number of mosquitoes captured inside a house; IR = infection rate; *Ny.* = *Nyssorhynchus*; Outdoor *N* = number of mosquitoes captured in the peridomestic environment (within 10 m of a main house entry); Pf = *Plasmodium falciparum*; Pv = *Plasmodium vivax*; N/A = not applicable.

**Figure 3.**
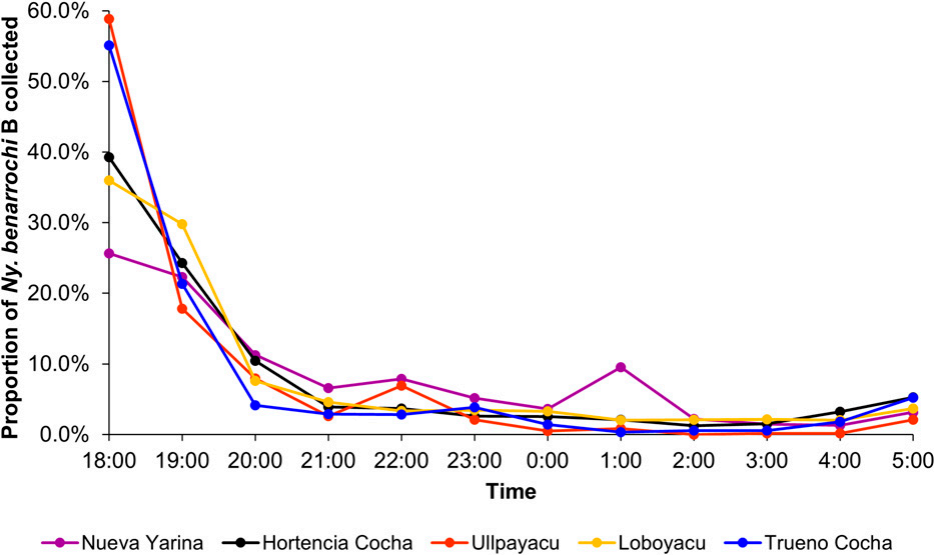
Average proportion of *Nyssorhynchus benarrochi* B collected per hour for all localities except Washientza, where *Ny. benarrochi* B was not collected.

Among the six main collection communities, 61.5% of all Anophelinae were captured outdoors (4,826/7,844). By community, most (five/six) had more outdoor specimens captured; however, in Washientza, the outdoor and indoor collection sizes were the same (*N* = 54) and all the samples were *Ny. darlingi* (Figure 2). Focusing exclusively on the biting pattern of *Ny. benarocchi* B because of its high abundance, the peak (average proportion/hour) occurred between 18:00 and 20:00, with a small peak detected at 01:00 only in Nueva Yarina (Figure 3). The highest HBRs for *Ny. benarrochi* B were in Loboyacu (592.8 bites/person/night) and Hortencia Cocha (579.5 b/p/n), whereas the highest HBR for *Ny. darlingi* was 32 b/p/n in Nueva Yarina (Table 2).

### *Plasmodium* infection.

Six ELISA pools of mosquitoes were confirmed positive for *P. falciparum or P. vivax*, and calculations were based on the assumption of one infective mosquito per positive pool (Table 2; Supplemental Table 1). The real-time PCR assay confirmed four of six infected individuals from the positive ELISA pools. Of the four species identified, only *Ny. darlingi* and *Ny. benarrochi* B were infected with *Plasmodium*. Infection rates, calculated per mosquito species, varied among localities; that is, IRs for *Ny. darlingi* were 0.94% (1/106) Washientza, 4.44% (2/45) Hortencia Cocha, and 1.56% (2/128) Nueva Yarina. Infection rates for *Ny. benarrochi* B were 0.04% (1/2,371) Loboyacu and 0 for the other localities (Table 2). Time of the collection of the six infected mosquitoes ranged across the night (Supplemental Table 1), and most (five/six) were collected outdoors. The infected specimen collected indoors was *Ny. darlingi* in Nueva Yarina (21:00–22:00), with *P. falciparum*.

Nueva Yarina and Hortencia Cocha had the highest EIR/night (0.5) where *Ny. darlingi* was infected (two *P. falciparum* [Pf] in Nueva Yarina; one Pf and one *P. vivax* [Pv] in Hortencia Cocha). In Loboyacu, we calculated an EIR of 0.25/night where *Ny. benarrochi* B was infected with Pv and in Washientza, the same EIR (0.25/night) where *Ny. darlingi* was infected with Pv. No infected mosquitoes were detected in either the largest village, Ullpayacu, or in Trueno Cocha (Table 2).

### Human malaria case incidence.

Overall, from 2018 to 2020, fewer cases of both *P*. *falciparum* and *P. vivax* were registered in the six riverine villages by 2020 (Table 1). However, in four villages, Hortencia Cocha, Nueva Yarina, Ullpayacu, and Trueno Cocha, *P. falciparum* cases increased from 2018 to 2020 at the same time as *P. vivax* cases decreased (except in Trueno Cocha). The overall proportion of *P. falciparum* from 2018 to 2020 was 29%, with *P. vivax* responsible for ∼71% (Table 1).

### Data management.

All anopheline individuals sequenced for the DNA COI barcode region (*N* = 21) have been deposited in GenBank. All sample results will be deposited in VectorBase^48^ pending publication.

## DISCUSSION

Entomological inoculation rate results greater than 0/night in four/six of the study villages in Datem del Marañon study were higher than generally reported for the dry season in Loreto (∼June–October), when both mosquito abundance and malaria cases trend lower.^7^^,^^49^ On the other hand, spatial heterogeneity among villages combined with occupation-related travel can lead to high EIR rates for *Ny. darlingi* even during the dry season.^50^ A striking result from this study is that in these riverine villages in Datem del Marañon both *Ny. darlingi* and *Ny. benarrochi* B were infected with *Plasmodium*, indicating that both are involved in local transmission. These data also support the role of *Ny. darlingi* as a primary vector in Loreto because even at low numbers and a relatively low biting rate (especially in contrast to the overwhelming abundance and high biting rate of *Ny. benarrochi* B in five/six villages), the EIR values for both species were in the same range.

Previous reports in Loreto have demonstrated that *Ny. benarrochi* (presumed to be *Ny. benarrochi* B) is both anthropophilic and abundant^19^^,^^20^ and can be infected.^21^ Importantly, we note that in these Datem del Marañon villages, the peak biting time of *Ny*. *benarrochi* B is early evening (∼18:00–20:00), a time when studies in many malaria endemic regions have shown that inhabitants are active, unprotected by bed nets, and vulnerable to transmission.^41^^,^^51^^,^^52^ Furthermore, most (five/six) infected mosquitoes were collected outdoors and were biting throughout the night. Such behavior patterns were recorded in Loreto previously for *Ny. darlingi*,^53^^,^^54^ although this species appears to revert to indoor biting when insecticide pressure is reduced.^55^ Biting behavior of *Ny*. *darlingi* is extremely plastic and depends on a wide array of microgeographic environmental conditions such as house location, forest cover, previous and current insecticide use, and human behavior.^56^^,^^57^

The current study, together with data from Colombia and Brazil,^24^^,^^26^^,^^27^ indicates that the role of *Ny*. *benarrochi* B in malaria transmission remains locally and regionally relevant, and additional studies on behavior and ecology are warranted. One limitation of this study is that collections were undertaken only in August during the dry season, generally the time of year in Amazonian Peru when both mosquito abundance and malaria cases are lower. Another limitation is that only two 12-hour collections were conducted per village for the main study. This collection is not representative of the common transmission pattern in Amazonian Peru, which is seasonal, high during the rainy season and low during the dry season.^41^ On the other hand, these data provide an entomological snapshot of *Plasmodium* infectivity in multiple villages in a region that has been neglected.

A study that analyzed 697,916 malaria cases in Peru between 2000 and 2017 determined that the highest mean API of *P. falciparum* occurred in Datem del Marañon (*M* = 26.2; SD = 25.9), whereas the highest API for *P. vivax* (*M* = 50.3, SD = 35.5) was in Loreto province.^36^ Despite the much smaller sample size in our study from six villages in Datem del Marañon (2018–2020), our findings also demonstrate an elevated proportion of *P. falciparum* of 28.99% (933/3,218) of the malaria cases (Table 1), in contrast to an average 25% of malaria cases in Peru.^11^^,^^12^ It is also noteworthy that subsequently, in 2021–2022, the district of Andoas was categorized as very high risk for *P. vivax* and *P. falciparum* malaria transmission (in Peru, an API > 50), with Pastaza in the same category for *P. vivax* but slightly lower for *P. falciparum* (high risk, API 10.00–49.99).^1^

Although we detected only three specimens of *Ny. tadei* from one village (Triunfo), it is the first confirmed report of this species in Datem del Marañon. This species belongs to the broadly distributed Amazonian Oswaldoi-Konderi complex (*Nyssorhynchus oswaldoi s.s.*, *Nyssorhynchus oswaldoi* A, *Nyssorhynchus oswaldoi* B, *Nyssorhynchus konderi*, and *Ny. tadei*) (Saraiva and Scarpassa^47^ and references therein). However, *Ny. tadei* (as *Nyssorhynchus sp. nr. konderi*) is known from Loreto, Peru,^41^^,^^58^^,^^59^ and is hypothesized to be allopatric in Loreto to the north and Madre de Dios to the south.^58^ In contrast, only a single specimen of *Ny. konderi* has been confirmed with molecular markers from the village of Salvador on the Napo River northwest of Iquitos, Loreto.^59^ Several earlier studies reported the presence of *Nyssorhynchus oswaldoi* s.l. in Peru, where it has been considered an effective malaria vector.^19^^,^^60^^,^^61^

In conclusion, in the malaria endemic province of Datem del Marañon, Amazonian Peru, *Ny. benarrochi* B was infected by *P. vivax* in Loboyacu and *Ny. darlingi* was infected by both *P. falciparum *and* P. vivax* in several villages. Although the risk to local human inhabitants of being bitten is highest during the early evening, risk of transmission was found to be throughout the night, mainly but not exclusively outdoors. As work toward malaria eradication in Peru moves forward with programs such as MZP, we recommend that remote and relatively understudied parts of Peru be a particular focus of attention and intervention.

## Supplemental Material


Supplemental materials
