# Supplementary material for: Natural Infection of Nyssorhynchus darlingi and Nyssorhynchus benarrochi B with Plasmodium during the Dry Season in the Understudied Low-Transmission Setting of Datem del Marañon Province, Amazonian Peru
Source: Am J Trop Med Hyg. 2023 Jun 26;109(2):288–95. doi: 10.4269/ajtmh.23-0058 (PMC10397451; doi:10.4269/ajtmh.23-0058)
Supplement: Supplementary file 1 [file tpmd230058.SD1.pdf]

Supplementary Material

Natural infection of *Nyssorhynchus darlingi* and *Nyssorhynchus benarrochi* B with *Plasmodium* during the dry season in the understudied low transmission setting of Datem del Marañon Province, Amazonian Peru

Jan E. Conn,[1,2,*] Sara A. Bickersmith,[1] Marlon P. Saavedra,[3] Juliana A. Morales,[3] Freddy Alava,[4] Gloria A. Diaz Rodriguez,[5] Clara R. del Aguila Morante,[5] Carlos G. Tong Rios,[5] Carlos Alvarez-Antonio,[6] Jesus M. Daza Huanahui,[7] Joseph M. Vinetz,[3,8,9,10,11] and Dionicia Gamboa.[3,8,11,*]

Table S1. Summary of *Plasmodium*-positive Anophelinae ELISA pools tested by real-time PCR from Datem del Marañon, 2019.

**Locality**	**Collection Date**	**HLC Location**	**Time (h)**	**Species**	**ELISA *Plasmodium* species**
Nueva Yarina	8/3/2019	Indoor	21:00 – 22:00	*Ny. darlingi*	*P. falciparum*
Nueva Yarina	8/3/2019	Outdoor	23:00 – 00:00	*Ny. darlingi*	*P. falciparum*
Hortencia Cocha	8/3/2019	Outdoor	18:00 – 19:00	*Ny. darlingi*	*P. vivax*
Hortencia Cocha	8/4/2019	Outdoor	05:00 – 06:00	*Ny. darlingi*	*P. falciparum*
Washientza	8/6/2019	Outdoor	19:00 – 20:00	*Ny. darlingi*	*P. vivax*
Loboyacu	8/7/2019	Outdoor	23:00 – 00:00	*Ny. benarrochi B*	*P. vivax*
